# Interleukin 1 receptor antagonist relation to cardiovascular disease risk in patients with rheumatoid arthritis

**DOI:** 10.1038/s41598-022-18128-5

**Published:** 2022-08-11

**Authors:** Cristina Almeida-Santiago, Juan Carlos Quevedo-Abeledo, Vanesa Hernández-Hernández, Antonia de Vera-González, Alejandra Gonzalez-Delgado, Miguel Ángel González-Gay, Iván Ferraz-Amaro

**Affiliations:** 1 Servicio de Reumatología, Hospital Universitario Dr. Negrín, Las Palmas de Gran Canaria, Spain; 2 Servicio de Reumatología, Hospital Universitario de Canarias, Tenerife, Spain; 3 Servicio de Laboratorio Central, Hospital Universitario de Canarias, Tenerife, Spain; 4 Division of Rheumatology, Hospital Universitario Marqués de Valdecilla, Santander, Spain; 5 Epidemiology, Genetics and Atherosclerosis Research Group on Systemic Inflammatory Diseases, Hospital Universitario Marqués de Valdecilla, IDIVAL, Santander, Spain; 6 Cardiovascular Pathophysiology and Genomics Research Unit, School of Physiology, Faculty of Health Sciences, University of the Witwatersrand, Johannesburg, South Africa; 7 Departamento de Medicina Interna, Universidad de La Laguna, Tenerife, Spain; 8 Division of Rheumatology, Hospital Universitario de Canarias, Tenerife, Spain

**Keywords:** Cardiovascular biology, Immunology, Biomarkers, Rheumatology

## Abstract

Interleukin (IL) 1, and its family member, IL-1 receptor antagonist (IL-1ra), are involved in the pathogenesis and inflammation perpetuation of patients with rheumatoid arthritis (RA). Besides, IL-1 has been linked to an increased risk and greater severity of cardiovascular (CV) disease. We aimed to study if IL-1ra is related to the CV manifestations—including lipid pattern and insulin resistance or subclinical atherosclerosis—that accompanies the disease in a large series of patients with RA. Cross-sectional study that encompassed 430 patients with RA. Serum IL-1ra levels were assessed. A multivariable analysis was performed to analyze the relation of IL-1ra to subclinical carotid atherosclerosis, and to traditional CV factors including a complete lipid molecules profile and insulin resistance or beta cell function indices. Body mass index, abdominal circumference, and the presence of obesity were significantly and positively associated with circulating IL-1ra. Similarly, erythrocyte sedimentation rate, and disease activity scores were significantly related to higher IL-1ra serum levels after adjustment for confounders. Neither carotid intima-media thickness nor the presence of carotid plaque were associated with serum levels of IL-1ra. However, after multivariable analysis circulating IL-1ra was independently and positively associated with higher serum levels of total cholesterol, triglycerides, and apolipoproteins B and C-III. Similarly, IL-1ra was related to higher levels of beta-cell function in the univariable analysis, although, in this case, significance was lost after adjustment. Among patients with RA, IL-1ra is associated with both disease activity and several traditional CV risk factors such as obesity and the presence of higher lipid levels. Our findings suggest that IL-1ra can represent a link between the inflammation and the CV disease risk that are present in patients with RA.

## Introduction

Interleukin (IL) 1 is a highly potent proinflammatory mediator that is important in immune defense and in immune-mediated disease. IL-1 encompasses two distinct cytokines, IL-1α and IL-1β, that signal via the same receptor, IL-1 receptor type 1. A related IL-1 family member, IL-1 receptor antagonist (IL-1ra), serves as an inhibitor that competes with both cytokines for binding to the receptor^1^.

There is ample evidence on the role of IL-1 in the development, pathogenesis, and inflammation perpetuation of patients with rheumatoid arthritis (RA)^2^. It is believed that a currently unknown antigenic trigger activates, among others, the production of IL-1 in the joint by macrophages, that eventually further produce or secrete additional cellular messengers such as the proteoglycans and proteases that can lead to the formation of the pannus, which accumulates in the joints. Destructive enzymes can then go on to destroy cartilage and ultimately degrade and erode bone. The role of IL-1 in RA or other inflammatory joint diseases has been supported by the fact that three therapies that target this cytokine (anakinra, canakinumab and rinolacept) have shown efficacy in clinical studies. Similarly, IL-1 cytokines have been associated with the pathogenesis of cardiovascular disease (CVD). In this sense, IL-1 has been found to represent a key pathogenetic mechanism in the formation, progression, and complication of atherosclerosis and the myocardial response to ischemic and nonischemic injury^3^. Furthermore, as in RA, IL-1 targeted therapies have already been shown to improve CV outcomes in clinical trials in patients with or at risk for myocardial infarction, heart failure, and recurrent pericarditis^4,5^.

Undoubtedly, rheumatoid arthritis (RA) has been associated with a greater presence of subclinical atheromatosis and a high incidence of CV events^6^. This accelerated CV disease in RA is believed to be due to the inflammatory activity that accompanies the disease^7^. However, the exact pathophysiological mechanisms or which specific inflammatory molecules are involved in this increased CV disease of RA are unknown.

In the present study, we have evaluated serum IL-1ra levels in a large series of RA patients who underwent evaluation for subclinical carotid atherosclerosis. In addition, a complete lipid pattern and indices of insulin resistance were measured. Our objective was to study how IL-1ra is related to CV risk factors such as dyslipidemia and insulin resistance, and to the subclinical atherosclerosis that is present in the disease.

## Material and methods

### Study participants

This was a cross-sectional study that included 430 consecutively recruited patients with RA. All of them were 18 years old or older and fulfilled the 2010 ACR/EULAR classification criteria^8^. They had been diagnosed by rheumatologists and were periodically followed-up at rheumatology outpatient clinics. For the purpose of inclusion in the present study, the duration of RA disease was required to be ≥ 1 year. Since glucocorticoids are often used in the treatment of RA, patients taking prednisone or an equivalent dose ≤ 10 mg / day were allowed to participate. Patients were excluded if they had a history of myocardial infarction, angina, stroke, a glomerular filtration rate < 60 ml/min/1.73 m^2^, a history of cancer or any other chronic disease such as hypothyroidism, heart or respiratory diseases, nephrotic syndrome, as well as evidence of active infection.

### Data collection and laboratory assessments

Individuals included in the study completed a CV risk factors and medications use questionnaire and underwent a physical examination. Body-mass index –BMI– (the weight in kilograms divided by the square of the height in meters), abdominal circumference, and systolic and diastolic blood pressure were assessed under standardized conditions. Information regarding smoking status, diabetes and hypertension was obtained from the questionnaire. Medical records were reviewed to ascertain specific diagnoses and medications. Disease activity in patients with RA was measured using the Disease Activity Score (DAS28) in 28 joints^9^, the Clinical Disease Activity Index (CDAI)^10^ and the Simple Disease Activity Index (SDAI)^11^. Disease disability was measured through the Health Assessment Questionnaire (HAQ) score^12^. Cholesterol, triglycerides, and HDL-cholesterol were measured using the enzymatic colorimetric assay. LDL-cholesterol was calculated using the Friedewald formula. A standard technique was used to measure the erythrocyte sedimentation rate (ESR) and high-sensitivity C-reactive protein (CRP). The homeostatic model assessment (HOMA) method was performed to determine IR. Briefly, the HOMA model enabled an estimate of insulin sensitivity (%S) and β-cell function (%B) from fasting plasma insulin, C peptide, and glucose concentrations. In this study we used HOMA2, the updated-computer HOMA model^13^. Human IL-1ra was measured using an enzyme-linked immunosorbent assay –ELISA– for its quantitative detection (Biovendor, Czech Republic). Both intra and inter-coefficients of variability are < 10% for this assay. Blood samples were taken fasting.

### Carotid ultrasound assessment

Carotid ultrasound examination was used to assess carotid intima media thickness (cIMT) in the common carotid artery and to detect focal plaques in the extracranial carotid tree in patients with RA^14^. A commercially available scanner, the Esaote Mylab 70 (Genoa, Italy), equipped with a 7–12 MHz linear transducer and an automated software-guided radiofrequency technique, Quality Intima Media Thickness in real-time (QIMT, Esaote, Maastricht, Holland), was used for this purpose. As previously reported^14^, based on the Mannheim consensus, plaque criteria in the accessible extracranial carotid tree (common carotid artery, bulb and internal carotid artery) were defined as follows: a focal protrusion in the lumen measuring at least cIMT > 1.5 mm; a protrusion at least 50% greater than the surrounding cIMT; or arterial lumen encroaching > 0.5 mm^15^.

### Statistical analysis

Demographic and clinical characteristics in patients with RA were described as mean (standard deviation) or percentages for categorical variables. For non-normally distributed continuous variables, data were expressed as median and interquartile range (IQR). Multivariable linear regression analysis, adjusting for confounders, was assessed to analyze the association between disease-related data and IL1-ra serum levels. Confounding variables were selected from demographics and traditional CV risk factors if they had a *p* value lower than 0.20 in the univariable relationship with IL1-ra. Besides, any variable that because of clinical reasons was considered as confounder was included in the multivariable analysis. Beta coefficients of the linear regression analyses were calculated as non-standardized coefficients. Collinearity in the multivariable regression models was checked through the calculation of the variance inflation factor (a variance inflation factor higher than 10 was considered a violation of collinearity). All the analyses used a 5% two-sided significance level and were performed using Stata software, version 17/SE (StataCorp, College Station, TX, USA). *P*-values < 0.05 were considered statistically significant.

### Ethics approval and consent to participate

The study protocol was approved by the institutional review committees at Hospital Universitario de Canarias and Hospital Universitario Doctor Negrín, and all subjects provided written informed consent (approval no. 2019–452-1). All research was performed in accordance with relevant guidelines/regulations and in accordance with the Declaration of Helsinki.

## Results

### Demographic and disease-related data

A total of 430 patients with RA were included in this study. Demographic- and disease-related characteristics of the participants are shown in Table 1. The mean age was 55 ± 10 years and 81% of the patients were women. Traditional CV risk factors were common. In this regard, 22% were current smokers, 13% had type 2 diabetes, 32% were considered obese (BMI equal to or greater than 30 kg/m^2^) and 34% had hypertension. Additionally, 32% of the patients were taking statins at the time of the study. The mean cIMT was 696 ± 131 microns, and 42% of the patients had carotid plaques. Full lipid profile and insulin resistance indices are additionally described in Table 1.

**Table 1:** Demographics, cardiovascular risk factors, and disease related data in RA patients.

	Rheumatoid arthritis
	(n = 430)
Age, years	55 ± 10
Female, n (%)	350 (81)
BMI, kg/m2	28 ± 5
Abdominal circumference, cm	97 ± 13
Cardiovascular data	
**CV risk factors, n (%)**	
Current smoker	93 (22)
Obesity	137 (32)
Hypertension	148 (34)
Diabetes Mellitus	54 (13)
Dyslipidemia	343 (80)
Statins, n (%)	139 (32)
**Analytical**	
Total cholesterol, mg/dl	205 ± 38
Triglycerides, mg/dl	147 ± 86
HDL-cholesterol, mg/dl	57 ± 15
LDL-cholesterol, mg/dl	120 ± 34
LDL:HDL cholesterol ratio	2.27 ± 0.93
Non-HDL cholesterol, mg/dl	149 ± 39
Lipoprotein (a), mg/dl	34 (11–107)
Apolipoprotein A1, mg/dl	173 ± 31
Apolipoprotein B, mg/dl	106 ± 26
Apo B:Apo A ratio	0.63 ± 0.24
Apolipoprotein C3, mg/dl	4.8 (2.2–8.7)
Glucose, mg/dl	95 ± 24
Insulin, µU/ml	8.6 (5.5–15.1)
C-peptide, ng/ml	2.5 (1.6–4.0)
HOMA2-IR	1.09 (0.7–2.0)
HOMA2-S%	92 (51–142)
HOMA2-B%-C-peptide	162 ± 77
**Disease related data**	
Disease duration, years	8 (4–15)
CRP at time of study, mg/l	2.7 (1.3–6.1)
ESR at time of study, mm/1st hour	18 (7–32)
IL-1ra, pg/ml	390 (14–2004)
Positive rheumatoid factor, n (%)	303 (72)
Positive ACPA, n (%)	253 (65)
DAS28-ESR	3.13 ± 1.35
DAS28-CRP	2.73 ± 1.08
SDAI	12 (7–19)
CDAI	8 (4–14)
History of extraarticular manifestations, n (%)	38 (10)
Erosions, n (%)	166 (43)
**Current drugs, n (%)**	
Prednisone	155 (36)
Prednisone doses, mg/day	5 (3–5)
NSAIDs	194 (45)
DMARDs	373 (87)
Methotrexate	316 (73)
Leflunomide	94 (22)
Hydroxychloroquine	45 (18)
Salazopyrin	28 (7)
Anti-TNF therapy	83 (19)
Tocilizumab	23 (5)
Rituximab	7 (2)
Abatacept	12 (3)
JAK inhibitors	20 (5)
Baricitinib	6 (1)
Tofacitinib	11 (3)
**Carotid ultrasound**	
cIMT, microns	696 ± 131
Carotid plaque, n (%)	180 (42)

Data represent mean ± SD or median (IQR) when data were not normally distributed.

CV: cardiovascular; LDL: low-density lipoprotein; HDL: high-density lipoprotein; CRP: C reactive protein. NSAID: Nonsteroidal anti-inflammatory drugs; DMARD: disease-modifying antirheumatic drug. TNF: tumor necrosis factor; Obesity; ESR: erythrocyte sedimentation rate. BMI: body mass index; DAS28: Disease Activity Score in 28 joints. ACPA: Anti-citrullinated protein antibodies. CDAI: Clinical Disease Activity Index; SDAI: Simple Disease Activity Index. IL1-ra: Interleukin 1 receptor antagonist; cIMT: carotid intima media thickness.

IL1-ra serum levels were 390 (IQR 14-2004) pg/ml. The median duration of the disease in this series of patients with RA was 8 (IQR 4-15) years. Seventy-two percent of patients were positive for rheumatoid factor, and 65% for anti-citrullinated protein antibodies. Disease activity measured by DAS28-ESR was 3.1 ± 1.4. Regarding this, 40% of the patients met the definitions of remission, and 18% and 42% were in the low, moderate/high disease activity categories, respectively. Thirty-six percent of the patients were being treated with prednisone and 87% were taking at least one conventional DMARD in any of its types, methotrexate being the most widely used (73%). Nineteen percent of the patients were receiving anti-tumor necrosis factor therapies. The frequency of use of other treatments and historical disease related data are shown in Table 1.

### Association of disease characteristics and cardiovascular risk factors with IL-1ra

The relation of CV risk factors and disease related data to IL-1ra (as dependent variable) is shown in Table 2. BMI (beta coefficient –coef. − 22 [95% confidence interval –CI– 14–30] pg/ml, *p* < 0.001), abdominal circumference (beta coef. 8 [95%CI 5–11) pg/ml, *p* < 0.001) and the presence of obesity (beta coef. 169 [95%CI 78–260] pg/ml, *p* < 0.001) were positively associated with circulating IL-1ra. Age showed a trend for a negative relationship to IL-1ra although in this case statistical significance was not reached. Besides, neither cIMT nor the presence of carotid plaque were associated with serum levels of IL-1ra.

**Table 2:** Demographics, cardiovascular risk factors, and disease related data in RA patients.

	IL1-Ra, pg/ml			
	Beta coef. (95% CI), *p*			
	Univariable		Multivariable	
Age, years	− 4 (− 8–0.2)	0.059		
Female, n (%)	− 4 (− 119–111)	0.95		
BMI, kg/m2	22 (14–30)	** < 0.001**		
Abdominal circumference, cm	8 (5–11)	** < 0.001**		
**Cardiovascular data**				
**CV risk factors, n (%)**				
Current smoker	− 52 (− 153–49)	0.31		
Obesity	169 (78–260)	** < 0.001**		
Hypertension	− 3 (− 91–86)	0.95		
Diabetes Mellitus	93 (− 34–221)	0.15		
Dyslipidemia	− 14 (− 114–86)	0.78		
Statins, n (%)	22 (− 69–113)	0.63		
**Disease related data**				
Disease duration, years	− 5 (− 9–− 0.02)	**0.042**	− 3 (− 8–1)	0.18
CRP at time of study, mg/l	1 (− 2–4)	0.41		
ESR at time of study, mm/ 1º hour	2 (0.09–5)	**0.042**	**3 (0.2–5)**	**0.034**
Rheumatoid factor, n (%)	− 91 (− 188–7)	0.069	− 58 (− 156–40)	0.24
ACPA, n (%)	− 51 (− 151–50)	0.32		
DAS28-ESR	60 (28–2)	** < 0.001**	**56 (25–88)**	**0.001**
DAS28-PCR	69 (30–108)	**0.001**	**59 (20–98)**	**0.003**
SDAI	3 (− 0.05–5)	0.055	2 (− 0.8–4)	0.17
CDAI	8 (2–13)	**0.005**	**8 (3–14)**	**0.003**
History of extraarticular manifestations, n (%)	26 (− 128–180)	0.74		
Erosions, n (%)	− 25 (− 116–67)	0.59		
**Current drugs, n (%)**				
Prednisone	− 12 (− 99–76)	0.79		
Prednisone doses, mg/day	− 18 (− 37–0.07)	0.058	− 11 (− 29–8)	0.26
NSAIDs	27 (− 58–113)	0.53		
DMARDs	− 86 (− 217–46)	0.20		
Methotrexate	− 56 (− 154–42)	0.26		
Leflunomide	− 13 (− 114–87)	0.79		
Hydroxychloroquine	− 137 (− 268–− 6)	**0.040**	− 125 (− 253–3)	0.055
Salazopyrin	− 98 (− 258–61)	0.23		
Anti-TNF therapy	− 18 (− 127–90)	0.74		
Tocilizumab	141 (− 35–316)	0.12	100 (− 71–271)	0.25
Rituximab	− 94 (− 496–309)	0.65		
Abatacept	− 28 (− 284–229)	0.83		
JAK inhibitors	− 125 (− 318–68)	0.20		
Baricitinib	− 201 (− 562–158)	0.27		
Tofacitinib	− 128 (− 384–128)	0.33		
**Carotid ultrasound**				
cIMT, microns	− 264 (− 605–78)	0.13	− 141 (− 535–253)	0.48
Carotid plaque, n (%)	− 73 (− 159–13)	0.095	− 23 (− 118–71)	0.63

Data represent mean ± SD or median (IQR) when data were not normally distributed.

CV: cardiovascular; CRP: C reactive protein; ESR: erythrocyte sedimentation rate. NSAID: Nonsteroidal anti-inflammatory drugs; DMARD: disease-modifying antirheumatic drug. TNF: tumor necrosis factor; BMI: body mass index; DAS28: Disease Activity Score in 28 joints. ACPA: Anti-citrullinated protein antibodies; cIMT: carotid intima media thickness. CDAI: Clinical Disease Activity Index; SDAI: Simple Disease Activity Index. Adjustment is performed for age, diabetes, BMI and waist circumference.

Significant p values are depicted in bold.

Regarding disease-related data, several significant associations were found, after multivariate analysis, for acute phase reactants and disease activity scores. For example, ESR, but not CRP, and DAS28-ESR, DAS 28-CRP and CDAI were significant related to IL-1ra after adjustment for confounders (Table 2). This was not the case for other disease characteristics such as the presence of rheumatoid factor or anti-citrullinated protein antibodies, or the use of several treatments. Only the use of hydroxychloroquine disclosed a negative relation to IL-1ra although statistical significance was not achieved (beta coef. − 125 [95%CI − 253–3] pg/ml, *p* = 0.055) (Table 2).

### Relationship of IL1-ra with the lipid profile and the indices of insulin resistance

The potential influence of IL-1ra serum levels (as independent variable) on lipid pattern molecules and insulin resistance indices is illustrated in Table 3. After multivariable analysis, IL1-ra was associated with higher serum levels of total cholesterol, triglycerides, non-HDL cholesterol, and apolipoprotein B and C-III. Remarkably, this was found after adjustment for age, diabetes, BMI and waist circumference (Table 3).

**Table 3:** IL1-ra relation to lipid profile and insulin resistance indices.

		IL-1ra ng/ml, beta coef. (95% CI)	
		Univariable	Multivariable
**Lipid pattern**			
Total cholesterol, mg/dl	206 ± 38	**11 (1–21), 0.025**	**11 (0.5–22), 0.039**
Triglycerides, mg/dl	147 ± 86	**45 (23–68), < 0.001**	**31 (8–54), 0..008**
HDL-cholesterol, mg/dl	57 ± 15	− 3 (− 7–0.7), 0.19	− 0.4 (− 5–3), 0.87
LDL-cholesterol, mg/dl	120 ± 34	5 (− 3–14), 0.23	5 (− 4–15), 0.28
LDL:HDL cholesterol ratio	2.20 ± 1.73	0.17 (− 0.31–0.65), 0.49	0.12 (− 0.39–0.64), 0.63
Non-HDL cholesterol, mg/dl	149 ± 39	**15 (5–25), 0.004**	**11 (0.9–22), 0.034**
Lipoprotein (a), mg/dl	34 (11–107)	3 (− 18–24), 0.76	4 (− 18–26), 0.73
Apolipoprotein A1, mg/dl	173 ± 31	− 3 (− 11–5), 0.47	2 (− 7–10), 0.69
Apolipoprotein B, mg/dl	107 ± 43	**13 (0.7–25), 0.038**	**13 (0.4–26), 0.043**
Apo B:Apo A ratio	0.63 ± 0.24	**0.07 (0.01–0.13), 0.030**	0.05 (− 0.02–0.12), 0.13
Apolipoprotein C-III, mg/dl	4.8 (2.2–8.7)	**3 (2–5), < 0.001**	**3 (1–4), < 0.001**
Glucose homeostasis molecules and IR indices*			
Glucose, mg/dl	87 ± 14	**− 3 (− 6–0.5), 0.023**	**− 4 (− 7**–** − 0.7), 0.016**
Insulin, µU/ml	10 ± 9	2 (− 0.6–4), 0.15	0.09 (− 2–2), 0.94
C-peptide, ng/ml	2.9 ± 2.1	0.3 (− 0.3–0.9), 0.36	0.03 (− 0.003–0.05), 0.087
HOMA2-IR	1.0 (0.6–2.6)	0.2 (− 0.09–0.5), 0.19	− 0.007 (− 0.3–0.3), 0.96
HOMA2-S%	118 ± 76	− 18 (− 40–3), 0.10	2 (− 20–23), 0.86
HOMA2-B%-C-peptide	165 ± 76	**24 (138–44), 0.019**	15 (− 6–36), 0.16

In this analysis IL1-Ra is the independent variable. IL1-ra is shown in ng/ml.

IL1-ra relation to glucose homeostasis molecules and insulin resistance indices is only performed in non-diabetic patients and glucose < 110 patients (n = 338).

LDL: low-density lipoprotein; HDL: high-density lipoprotein. HOMA: homeostatic model assessment.

Adjustment is performed for age, diabetes, BMI and waist circumference. Significant p values are depicted in bold.

The analysis of glucose homeostasis molecules and indices of insulin resistance was only performed in patients with RA who were not diabetic and had a blood glucose lower than 110 mg/dl (Table 3). In this analysis, IL-1ra was associated with higher levels of beta cellfunction (HOMA2-B%) in the univariable analysis (beta coef. 24 [95%CI 138–4], *p* = 0.019). However, after full multivariable adjustment this relationship was lost (Table 3).

## Discussion

Our study is the largest in which IL-1ra serum levels have been analyzed in patients with RA. According to our results, IL-1ra may not only act as an acute phase reactant associated with disease activity, but is also related to some CV comorbidities such as BMI, abdominal circumference and the presence of dyslipidemia. These findings suggest that IL-1 can be involved in both the inflammation present in RA and in the CV comorbidity that accompanies this disease.

We have found in our work a clear relationship between body mass index and abdominal circumference and serum levels of IL-1ra. This is in agreement with the notion that adipose tissue can produce cytokines that have crucial roles in regulating metabolism and immune responses. In obese patients in the general population, it has been described that adipose tissue is responsible for low-grade inflammation through dysregulatory proinflammatory factors^16^. In obesity, the increased level of adipose tissue-derived IL-1 can result in low-grade inflammation, which is the main culprit of obesity-related complications, such as insulin resistance and liver fibrosis which can facilitate type 2 diabetes and atherosclerosis development^17^. Probably, and based on our findings, this relationship observed in the general population would be maintained in patients with RA.

A positive and significant relationship between IL-1ra and several molecules related to lipid metabolism such as total cholesterol, triglycerides and lipoproteins B and C-III was found in our study. This is in accordance with the knowledge that IL-1 has been suggested to directly modulate lipid metabolism by suppressing lipoprotein lipase activity^18^. However, we and others have shown an inverse relationship between inflammation and lipid levels in RA patients^19^. This does not appear to be the case for IL-1ra where this acute phase reactant is positively associated with higher levels of lipid molecules. However, the fact that most of the patients in our series had low and moderate disease activity and the cross-sectional design of our study does not allow definitive conclusions to be drawn about the influence of IL-1ra on the lipid pattern.

In pancreatic beta cells, IL-1 participates in cytokine-mediated apoptosis and mediates oxidative stress-induced suppression of insulin gene transcription^20^. Moreover, IL-1β decreases expression of the insulin receptor-1, inhibits glucose transporter translocation to the plasma membrane, and reduces insulin-stimulated glucose uptake and lipogenesis^21^. This has been supported by the fact that emerging clinical studies have shown that treatment with IL-1 receptor antagonists (anakinra) and IL-1β-specific antibodies (canakinumab) improved glucose metabolism and insulin secretion in patients with type 2 diabetes^22^. Our result of a relationship between IL-1ra and beta cell function, although only in univariable analysis, is in agreement with what was previously described in a healthy population.

We acknowledge the limitation that in our study we assessed IL1-ra but not IL-1α and IL-1β. However, the assessment of these two cytokines in serum has difficulties due to several factors^23^. In this sense, circulating IL-1α is usually absent because most of the IL-1α remains in the cytosol of cells in its precursor form, where it can function as an autocrine messenger. Similarly, plasma concentrations of IL-1β are usually below the limit of detection of the available assays (5 pg per milliliter)^23^. In addition, it is recommended to measure the production of IL-1β in peripheral blood mononuclear cells rather than in serum, since it is generally absent in the latter^24^. Furthermore, IL-1ra concentrations primarily reflect IL-1 production, so serum levels of the former are proportional to those of the latter^25^. Taking these considerations together, we believe that measuring IL-1ra in our work was a valid representation of global IL-1 metabolism.

The results shown in this study support the clinical and therapeutic relevance of the biological aspects of IL-1 in CV disease of RA. Three agents are currently available for pharmaceutical inhibition of IL-1: the recombinant IL-1 receptor antagonist (IL-1ra) anakinra; the anti-IL-1β antibody canakinumab; and the IL-1 “trap” fusion molecule rilonacept. In the first large-scale blinded and placebo-controlled, randomized clinical trial that targeted inflammation but not lipids, CANTOS (Canakinumab Anti-Inflammatory Thrombosis Outcome Study) supported the inflammatory hypothesis of atherosclerosis^26^. Additionally, the CANTOS results highlighted the utility of assessing both LDL-cholesterol and CRP, as targeting either of these biomarkers can reduce recurrent events. We believe that IL-1 inhibition can have a beneficial effect on CV disease and CV events in RA patients.

## Conclusion

In conclusion, in patients with RA, serum levels of IL1-ra are significantly associated with the clinical and inflammatory activity of the disease, as well as with different CV comorbidities such as dyslipidemia, beta cell function and obesity. Our findings therefore emphasize the beneficial effect that IL1 inhibition drugs can have both in controlling RA disease activity and the accelerated CV disease that these patients manifest.

## Data Availability

The data sets used and/or analyzed in the present study are available from the corresponding author upon request.

## Abbreviations

ACPA: Anti-citrullinated peptide/protein antibody
BMI: Body mass index
CDAI: Clinical disease activity index
CI: Confidence of interval
cIMT: Carotid intima-media thickness
CRP: C-reactive protein
CV: Cardiovascular
DAS28: Disease activity score in 28 joints
DMARD: Disease-modifying antirheumatic drug
ELISA: Enzyme-linked immunosorbent assay
ESR: Erythrocyte sedimentation rate
HAQ: Health assessment questionnaire
HDL: High-density lipoprotein
HOMA: Homeostatic model assessment
IL-1: Interleukin 1; IL-1ra: Interleukin 1 receptor antagonist
IQR: Inter quartile range
LDL: Low-density lipoprotein
LPL: Lipoprotein lipase
NSAID: Nonsteroidal anti-inflammatory drug
RA: Rheumatoid arthritis
SD: Standard deviation
SDAI: Simplified disease activity index
TNF-α: Tumor necrosis factor-α
